# Increased Risk of Acute Pancreatitis in Patients with Type 2 Diabetes: An Observational Study Using a Japanese Hospital Database

**DOI:** 10.1371/journal.pone.0053224

**Published:** 2012-12-27

**Authors:** Hisashi Urushihara, Masanori Taketsuna, Ying Liu, Eisei Oda, Masaki Nakamura, Shinichi Nishiuma, Rei Maeda

**Affiliations:** 1 Department of Pharmacoepidemiology, Graduate School of Medicine and Public Health, Kyoto University, Kyoto, Japan; 2 Asia-Pacific Statistical Sciences, Eli Lilly Japan K.K., Kobe, Hyogo, Japan; 3 Global Patient Safety Japan, Eli Lilly Japan K.K., Kobe, Hyogo, Japan; 4 Medical TOUKEI Corporation, Shinjuku-ku, Tokyo, Japan; 5 Business Development Department, Medical Data Vision Co., Ltd., Chiyoda-ku, Tokyo, Japan; University of Texas Health Science Center at San Antonio, United States of America

## Abstract

**Background:**

Increased risks of acute pancreatitis in patients with type 2 diabetes mellitus have been reported recently in several countries. We aimed to estimate the risks of acute pancreatitis in Japanese patients with diabetes mellitus.

**Methods/Findings:**

We examined a large-scale hospital administrative database consisting of one million patients in 16 secondary medical care hospitals, from 2003 to 2010. The incidence rates of acute pancreatitis were estimated with cohort design; the odds ratios associated with diabetes mellitus and other comorbid risk factors were estimated with separate case-control analyses.

In cohort analysis, the incidence of acute pancreatitis was higher in 14,707 diabetic patients than in 186,032 non-diabetic patients (4.75 vs. 1.65 per 1,000 patient-years) and increased in male patients and as age advanced. The adjusted odds ratio of acute pancreatitis in patients with diabetes mellitus was 1.86 (*P*<0.001) compared with non-diabetic patients in case-control analysis from 1,372 cases and 5,469 matched controls, which is consistent with the ones reported in previous studies. Alcoholism and gallstones were associated with a large increase in the risk of acute pancreatitis (adjusted odds ratio 13.40 and 14.29, respectively, *P*<0.001), although dyslipidemia was associated with significant risk reduction (adjusted odds ratio 0.62, *P*<0.001).

**Conclusions:**

This observational study ascertained the elevated incidence rates and risk of acute pancreatitis in Japanese patients with diabetes. The risk estimates in Japanese patients with diabetes were in agreement with the ones reported in previous studies, and the elevated risk of acute pancreatitis in patients with diabetes would be generalized in different locations/populations.

## Introduction

Acute pancreatitis (AP) is becoming increasingly prevalent in Japan as well as in Western countries [1]–[4]. The nationwide epidemiological survey of AP in the Japanese population revealed that its annual incidence in 2007 reached 57,560, with a prevalence of 45.1 per 100,000 people [5]. A variety of risk factors for AP have been established, including alcoholic consumption, gallstones, obesity, hypertriglyceridemia, viral hepatitis, chronic pancreatitis, as well as some medications and other risk factors [6], [7]. Some of these are also common complications and risk factors for type 2 diabetes mellitus (DM).The association of AP with type 2 DM was first suggested by a randomized controlled trial of fenofibrate in patients with type 2 DM [8]. During the follow-up in the placebo arm, a higher cumulative incidence of AP was reported in those patients in the placebo arm than in the general population estimates. The first observational study using a US healthcare claim database reported that patients with type 2 DM had an increased risk of AP compared with the non-DM population in 2009 [9]. On the other hand, the Japanese 2007 national survey reported that 11% of AP patients had DM as the most frequent comorbidity [5].

It should be considered of importance to quantify the background incidence and risk of AP in Japanese patients with DM for the purpose of appropriate clinical management of DM, since the Japanese have culture-specific differences in nutrition and hereditary factors predisposing them to DM compared to Western countries [10]. So far, there have been no studies investigating the risks of AP in Japanese patients with DM. Thus, we conducted an epidemiological study to estimate an AP risk associated with DM using a Japanese hospital administrative database.

## Methods

### Data source

This retrospective observational study was performed using a hospital-based composite database containing administrative data and laboratory values stored in hospital electronic information systems, which was constructed by Medical Data Vision Co., Ltd (Tokyo, Japan) and used for epidemiological research [11]. The source population of the database was derived from 16 secondary medical care hospitals with number of beds ranging from 20 to over 1,000 (with a mean of approximately 300), located in multiple districts. This database has aggregated the medical services of more than 1 million patients since the start of data collection in January 2003, and contains an anonymized patient identifier, gender, birth year, department, date of medical service, diagnosis codes, hospitalization, medical procedures and test orders, operations, prescriptions, and a standard set of laboratory values such as blood counts and chemistry. Age and gender distributions of the patients in the database are approximately similar to that of the national patient statistics in Japan [12]. The data collected between January 1, 2003 and December 31, 2010 was analyzed.

### Disease Definition

Disease criteria were defined according to the International Statistical Classification of Diseases and Related Health Problems 10^th^ Revision (2003 Version) (ICD10), which is used in hospital information systems for claim reimbursement within the Japanese national medical insurance scheme. Type 2 DM was identified with the following ICD10 codes: E11 (noninsulin dependent diabetes mellitus); E12 (malnutrition-related diabetes mellitus); E13 (other specified diabetes mellitus); and E14 (unspecified diabetes mellitus). Having a prescription history of antidiabetic medications is the second criteria, including oral antidiabetics, incretin products, and insulin and its deliverables. This criterion is to minimize possible contamination by the patients who were suspected DM because of the presence of glucose intolerance and tentative hyperglycemia, and administered examinations to rule out DM but coded with DM for the purpose of claim reimbursement [9]. Patients with type 1 DM (ICD10 code: E10) were excluded. Cases of AP were determined by diagnosis records of acute pancreatitis (K85). To exclude possibilities of tentative diagnosis for the purpose of claim reimbursements for examinations, AP occurrences were confined to patients satisfying the following criteria: 1) having claims for abdominal image tests including ultrasonography, plain X-ray, computed tomography, and magnetic resonance imaging within 3 days before and after the date of AP diagnosis; and 2) being hospitalized within a period of two weeks after the diagnosis date and for a duration of 3 days or more [6], [13]. Risk factors considered in this analysis included: obesity (E66); dyslipidemia (E78); alcoholic dependence syndrome (F10.2); gallstones (K80); obstruction of bile duct (K83.1); other pancreas diseases (K86.2 to 86.9); viral hepatitis B and C (B16, B17.0 to 17.1, B18.0 to 18.2); and surgeries for digestive system diseases, which were identified by claim codes for the national health insurance medical fee schedule [14]. Having autoimmune diseases, which is possibly associated with autoimmune pancreatitis such as sicca syndrome or Sjogren's syndrome, primary sclerosing cholangitis, and autoimmune hepatitis, was not considered for statistical adjustment [7].

### Study Design and Population

**Figure 1:**
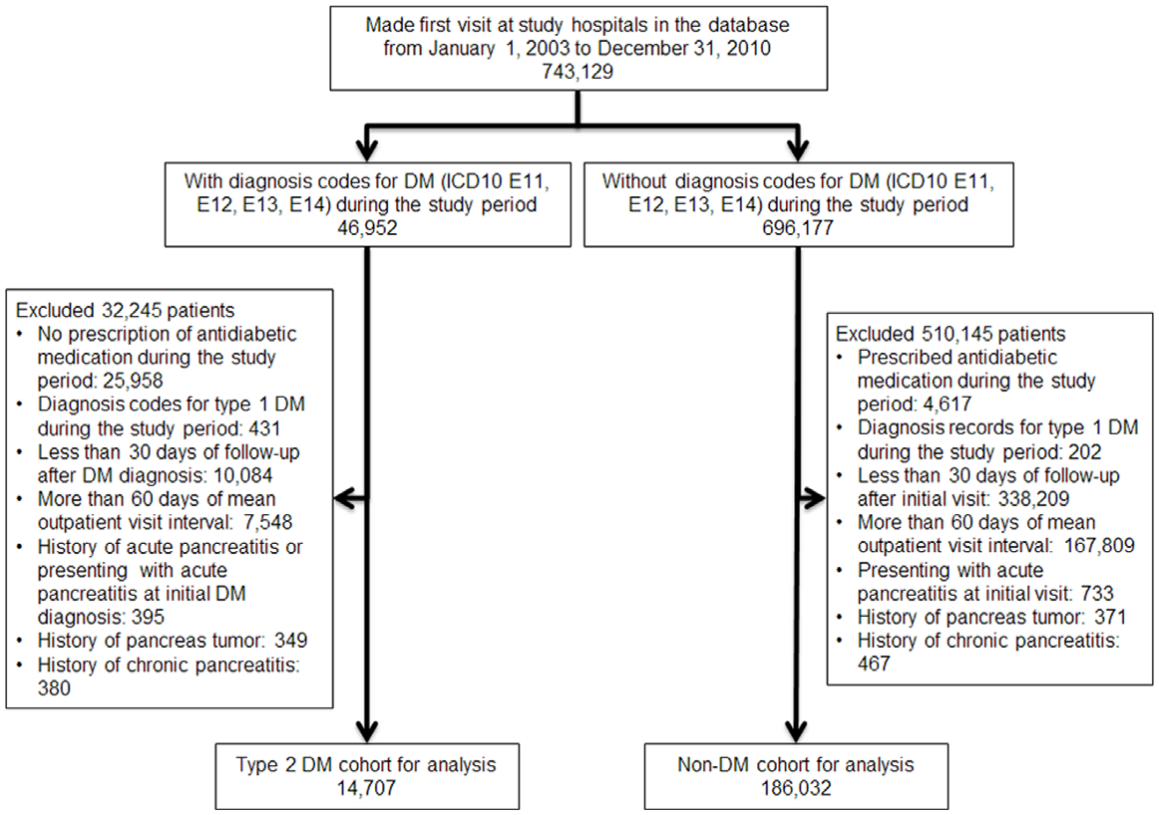
Patient selection criteria for cohort substudy of the risk of acute pancreatitis associated with type 2 diabetes mellitus. DM, diabetes mellitus; ICD10, International Statistical Classification of Diseases and Related Health Problems 10th Revision Version for 2003.

This study consisted of separate cohort- and case-control substudies. For both substudies, patients eligible for enrollment were aged 18 years or more at the hospital visit for which a claim for an initial visit fee was recorded during the study period (initial visit). A total of 743,129 patients made initial visits to the study hospitals during the period from January 1, 2003 to December 31, 2010 (Figure 1).

The first cohort substudy aimed to estimate absolute incidence of AP in the type 2 DM population. For this cohort substudy, 46,952 patients with the diagnosis codes for DM (E11 to E14) were screened for the above-defined criteria for type 2 DM; 25,958 patients with no prescription history of antidiabetic medications and 431 patients with type 1 DM were excluded. For the non-DM cohort, 696,177 patients without the diagnosis code for DM (E11 to E14) were screened; 4,617 patients having prescription records for antidiabetic medications and 202 patients with a diagnosis of insulin-dependent DM were excluded. Cohort patients were required to have follow-up periods of 30 days or more on the database from the diagnosis date of DM for the DM cohort and from the initial visit date for non-DM cohort (index visit) and mean visit intervals of 60 days or less as outpatients, in order to ensure continuity of follow-up in the study database for both cohorts. Patients with a history of pancreatic tumor (C25) and patients presenting with AP at the index visit to the study hospital were ineligible for the cohort substudy. Patients with a history of chronic pancreatitis (K86.0 and K86.1) were excluded since chronic pancreatitis is associated with development of type 2 DM and acute exacerbation of chronic pancreatitis is often miscoded as acute pancreatitis. Autoimmune pancreatitis (K86.1) is included in the definition of chronic pancreatitis adopted in this study, according to ICD-10 classification, and therefore excluded from the study population. Patients with a history of acute pancreatitis before the index visit were also excluded from the DM cohort.

The second case-control substudy primarily aimed at estimating odds ratios for AP in patients with type 2 DM compared with non-DM patients. A substantial portion of patients with an AP diagnosis in the database would have a great likelihood of presenting with symptoms of AP at the initial visit to the study hospital in the database. Patients who comprised these cohorts were required to have been free from the outcome of concern at study entry, subsequently leading to a substantial loss of AP cases in the cohort substudy. To secure a number of AP cases at initial appearance to the study hospitals for risk estimation, a separate case-control analysis was conducted using the same database. Thus, nested-case control design was not selected. For the case-control substudy, all of the patients meeting the disease criteria for AP were case candidates. Controls were selected from the patients who did not satisfy the AP diagnosis criteria. The exclusion criteria included: 1) having a diagnosis code for type 1 DM (E10); 2) a history of pancreatic tumor (C25); 3) a history of chronic pancreatitis (K86.0 and K86.1) and 4) having a diagnosis code for type 2 DM (E11 to E14) but no prescription records of the antidiabetic medications specified above (indeterminates). The remaining 1,375 AP cases were eligible for pair-matching. The cases and controls were pair-matched 1∶4 at a maximum according to hospital visit timing, gender, birth year, and geographical location. In sampling controls, the given visit date of a control was matched with the AP diagnosis date of a case (sampling date). Controls were required to have had the follow-up period of 30 days or more before the sampling date to secure sufficient verification time for disease histories. As a result, 1,372 cases were successfully matched with 5,469 controls.

### Ethics Statement

Because the data investigated in the present study were de-identified at the study hospitals before being incorporated into the Medical Data Vision automated hospital information database and retrieved from the database in an unlinked manner, the study was exempt from obtaining informed consent from individual patients according to the local ethical guidelines for epidemiological research. This study and the waiver of informed consent were approved by the Ethics Committee of the Japan Epidemiological Association [15].

### Statistical Analysis

Patient characteristics were summarized with descriptive statistics. Student t and Wilcoxon rank sum tests for continuous variables and chi-square tests for categorical variables were used to test differences in patient characteristics between the DM cohort and the non-DM cohort in the cohort substudy. The AP incidence rates in person-years were estimated as the number of acute pancreatitis cases divided by the total period at risk of AP in each cohort. The period at risk began on the earliest date of the diagnosis for patients with type 2 DM and on the initial visit date for non-DM patients and ended on the AP diagnosis date defined above or the date of the last hospital visit record in the database, whichever came earlier. The period at AP risk was censored at the first occurrence of pancreatic tumor or chronic pancreatitis after index visit. Crude relative risks of AP were calculated for the total eligible patients and by gender and age group, by comparison of the incidence rates between type 2 DM and non-DM cohorts. A hazard ratio for developing AP, adjusted for the risk factors in the cohort substudy, was estimated by a Cox regression model. In the case-control substudy, the AP risk for patients with type 2 DM was estimated as an odds ratio using conditional logistic regression, adjusted for concerned comorbidities. For statistical tests, a two-tailed significance level of 0.05 was used and multiplicity was not considered. Statistical analyses were performed with SAS 9.1.3. for Windows (SAS Institute Inc., Cary, NC, USA).

## Results

### Cohort substudy

The cohort substudy consisted of 14,707 eligible patients in the type 2 DM cohort and 186,032 patients in the non-DM cohort (Figure 1). The overall clinical picture of the cohort populations was illustrated with the distribution of diagnosis codes according to the ICD10 blocks in Table S1. The type 2 DM cohort had more frequent diagnosis codes in most of the disease classes. Gender- and age-distributions significantly differed between the cohorts; patients with type 2 DM were approximately 10 years older than non-DM patients (mean age ± SD 65.6±13.2 and 54.9±19.4, respectively, *P*<0.001) (Table 1) and there were more males in the type 2 DM cohort than in the non-DM cohort (61.4% and 43.3%, respectively, *P*<0.001). The mean period at risk was significantly longer in patients with type 2 DM (614.4±583.8 days) than non-DM patients (424.4±507.6 days, *P*<0.001 vs. type 2 DM). All the comorbid risk factors were more significantly prevalent in the type 2 DM cohort, including obesity, dyslipidemia, alcoholism, gallstones, biliary obstruction, other pancreas diseases, viral hepatitis, and surgery for digestive system diseases at baseline.

**Table 1:** Baseline characteristics of the type 2 DM and non-DM cohorts in the cohort substudy.

	Type 2 DM cohort	Non-DM cohort	*P* value*
**Number of subjects**	14,707	186,032	
**Female**	5,670 (38.6)	105,417 (56.7)	<0.001
**Age (years)**	65.6±13.2	54.9±19.4	<0.001**
**Period at risk (days)**	614.4±583.8	424.4±507.6	<0.001***
**(Min-max)**	2–2,815	2–2,827	
**Comorbidity**			
**Obesity**	92 (0.6)	247 (0.1)	<0.001
**Dyslipidemia**	6,478 (44.0)	14,093 (7.6)	<0.001
**Alcoholism**	27 (0.2)	142 (0.1)	<0.001
**Gallstones**	1,144 (7.8)	5,148 (2.8)	<0.001
**Biliary obstruction**	190 (1.3)	562 (0.3)	<0.001
**Other pancreas diseases excluding pancreatitis**	191 (1.3)	559 (0.3)	<0.001
**Hepatitis B and C**	697 (4.7)	3,285 (1.8)	<0.001
**Surgeries of digestive system diseases**	2,326 (15.8)	13,429 (7.2)	<0.001

DM, diabetes mellitus.

Data are presented as means ± SD or numbers with percent in parenthesis.

* Type 2 DM vs. non-DM. No mark indicates *P* values for Chi-square tests.

** *P* value for *t* test.

*** *P* value for Wilcoxon rank sum test.

We identified a total of 473 AP cases, 117 in the type 2 DM cohort (0.80%) and 356 in the non-DM cohort (0.19%). The incidence rate (IR) of AP in the type 2 DM cohort was 4.75 per 1,000 patient-years (95% CI 3.97–5.70) and 2.88-fold greater than in the non-DM cohort (IR 1.65 per 1,000 patient-years, 95% CI 1.49–1.83) (Table 2). The age-specific IR in patients with type 2 DM increased as age increased and was highest in the most elderly patient strata, those aged 80 years or more (10.60 per 1,000 patient-years, 95% CI 7.04–15.95). The crude rate ratio (RR) for AP in patients with type 2 DM increased with age and was highest for patients aged 70–79 years (crude RR 3.56, 95% CI 2.44–5.18) and for patients aged 80 years or older (3.56, 95% CI 2.21–5.75), compared with the non-DM peer groups. Based on a multivariate Cox proportional hazard model after controlling for gender, age group, comorbid risk factors including dyslipidemia, alcoholism, gallstones, biliary obstruction, other pancreatic diseases excluding pancreatitis, and surgeries for digestive system diseases, a significantly elevated hazard ratio (HR) for AP in patients with type 2 DM (HR 2.30, 95% CI 1.83–2.89, *P*<0.001) was also shown compared with the non-DM patients. Morbid obesity and viral hepatitis were excluded from this model because neither of them made a significant contribution to AP risk and therefore they were also omitted from subsequent analyses.

**Table 2:** Incidence and relative risks of acute pancreatitis estimated by gender, age for type 2 DM and non-DM cohorts: cohort substudy.

	Type 2 DM cohort				Non-DM cohort				
Strata	Number of subjects	Incident cases	Patient-years at risk	Incidence rate per 1,000 patient-years [95% CI]	Number of subjects	Incident cases	Patient-years at risk	Incidence rate per 1,000 patient-years [95% CI]	Crude rate ratio: Type 2 DM vs. non-DM [95% CI]
**All subjects**	14,707	117	24,615	4.75 [3.97, 5.70]	186,032	356	215,782	1.65 [1.49, 1.83]	2.88 [2.34, 3.55]
**Gender**									
**Female**	5,670	39	9,122	4.28 [3.12, 5.85]	105,417	172	127,319	1.35 [1.16, 1.57]	3.16 [2.24, 4.48]
**Male**	9,037	78	15,493	5.03 [4.03, 6.29]	80,615	184	88,463	2.08 [1.80, 2.40]	2.42 [1.86, 3.15]
**Age (years)**									
**18–39**	610	1	1,035	0.97 [0.14, 6.86]	50,627	51	44,183	1.15 [0.88, 1.52]	0.84 [0.12, 6.06]
**40–49**	1,091	3	2,019	1.49 [0.48, 4.61]	22,957	31	24,007	1.29 [0.91, 1.84]	1.15 [0.35, 3.76]
**50–59**	2,682	15	5,318	2.82 [1.70, 4.68]	28,372	55	36,495	1.51 [1.16, 1.96]	1.87 [1.06, 3.31]
**60–69**	4,253	34	8,182	4.16 [2.97, 5.82]	34,187	75	48,505	1.55 [1.23, 1.94]	2.69 [1.79, 4.03]
**70–79**	3,966	41	5,891	6.96 [5.12, 9.45]	30,177	81	41,405	1.96 [1.57, 2.43]	3.56 [2.44, 5.18]
**≥80**	2,105	23	2,170	10.60 [7.04, 15.95]	19,712	63	21,186	2.97 [2.32, 3.81]	3.56 [2.21, 5.75]

DM, diabetes mellitus.

### Case-control substudy

To salvage the substantial number of AP cases at initial hospital visits that could not be available for cohort analysis and to control for the difference in age- and gender- distribution between patients with DM and non-DM patients, a separate case-control analysis was conducted using the same database. The overall clinical picture of the 1,372 AP cases and 5,469 pair-matched controls were illustrated with the distribution of diagnosis according to the ICD10 classification in Table S2. Male patients were more prevalent than female patients in both cases and sampled controls (Table 3). Gallstones were the most frequently observed among the comorbidities under review (37.2%) in the cases and all the comorbidities except dyslipidemia (cases 10.2%, controls 13.3%) were more prevalent in the case population than the control population.

**Table 3:** Baseline characteristics in case-control substudy.

	Acute pancreatitis cases^a^		Matched controls^b^	
	Type 2 DM patients	Non-DM patients	Type 2 DM patients	Non-DM patients
**Number of subjects**	244	1,128	629	4,840
**Female**	83 (34.0)	499 (44.2)	188 (29.9)	2,130 (44.0)
**Age (years)**	68.5±12.8	61.7±18.8	66.5±12.5	61.5±18.6
**Comorbidity**				
**Obesity**	0	2 (0.2)	0	4 (0.1)
**Dyslipidemia**	44 (18.0)	96 (8.5)	251 (39.9)	476 (9.8)
**Alcoholism**	2 (0.8)	8 (0.7)	1 (0.2)	4 (0.1)
**Gallstones**	77 (31.6)	434 (38.5)	49 (7.8)	160 (3.3)
**Biliary obstruction**	34 (13.9)	113 (10.0)	10 (1.6)	19 (0.4)
**Other pancreas diseases excluding pancreatitis**	7 (2.9)	24 (2.1)	21 (3.3)	31 (0.6)
**Hepatitis B and C**	11 (4.5)	31 (2.7)	35 (5.6)	127 (2.6)
**Surgeries for digestive system diseases**	62 (25.4)	145 (12.9)	99 (15.7)	402 (8.3)

DM, diabetes mellitus.

Data are presented as means ± SD or numbers with percent in parenthesis. Characteristics presented at diagnosis for acute pancreatitis cases and at sampling visit for matched controls.

a n = 1,372.

b n = 5,469.

Based on univariate conditional logistic regression analysis, type 2 DM significantly increased the risk of AP (unadjusted odds ratio [OR] 1.72, 95% CI 1.46–2.04, *P*<0.001) (Table 4). Alcoholism, gallstones, biliary obstruction, pancreas diseases other than pancreatitis, and surgeries for digestive system diseases were comorbidities associated with significant increases in AP risk; however, dyslipidemia was associated with a significant decrease in AP risk (unadjusted OR 0.73, 95% CI 0.60–0.89, *P* = 0.002). After controlling for all these covariates in the multivariate model, the increase in AP risk for patients with type 2 DM remained significant (adjusted OR 1.86, 95% CI 1.51–2.29, *P*<0.001), suggesting its being an independent risk factor. The three strongest risk factors were biliary obstruction (19.23, 95% CI 11.55–32.04), gallstones (14.29, 95% CI 11.60–17.62), and alcoholism (13.40, 95% CI 4.27–42.04). An adjusted OR of pancreatic diseases excluding pancreatitis was similar to the one of type 2 DM (1.99, 95% CI 1.13–3.51). A decreased risk of AP with dyslipidemia remained significant (0.62, 95% CI 0.48–0.79); however, surgeries for digestive system diseases were no longer significantly associated with increased AP risk (*P* = 0.882).

**Table 4:** Univariate and multivariate conditional logistic regression for estimating acute pancreatitis risks in case-control substudy.

	Univariate model		Multivariate model	
Risk factors	Odds ratio [95% CI]	*P* value	Odds ratio [95% CI]^a^	*P* value
**Type 2 diabetes mellitus**	1.72 [1.46, 2.04]	<0.001	1.86 [1.51, 2.29]	<0.001
**Dyslipidemia**	0.73 [0.60, 0.89]	0.002	0.62 [0.48, 0.79]	<0.001
**Alcoholism**	8.00 [2.73, 23.41]	<0.001	13.40 [4.27, 42.04]	<0.001
**Gallstones**	15.07 [12.36, 18.36]	<0.001	14.29 [11.60, 17.62]	<0.001
**Biliary obstruction**	25.81 [16.48, 40.42]	<0.001	19.23 [11.55, 32.04]	<0.001
**Other pancreatic diseases excluding pancreatitis**	2.42 [1.54, 3.79]	<0.001	1.99 [1.13, 3.51]	0.017
**Surgeries for digestive system diseases**	1.80 [1.51, 2.15]	<0.001	1.02 [0.81, 1.28]	0.882

Cases and controls were matched for hospital visit timing, gender, birth year, and geographic location.

a Odds ratios were adjusted for all comorbidities in the table.

## Discussion

This retrospective observational study using a hospital database confirmed that type 2 DM was associated with a higher incidence of AP and the increased risk was approximately two-fold in Japanese patients. Our estimates of AP risks in patients with diabetes were consistent in terms of magnitude with the ones reported in other previous studies using automated health care databases in the US, the UK, and Taiwan [9], [16]–[19]. After adjustment for all the comorbidities of concern, gallstones, biliary obstruction, and alcoholism were confirmed as the strongest independent risk factors for developing AP, whereas dyslipidemia was associated with a significant risk reduction.

The incidence rates of AP in patients with type 2 DM were consistently higher than non-DM patients regardless of gender and age. The overall incidences for patients with DM and for non-DM patients were very similar to those reported in previous studies using claims databases in the US and Taiwan (Table S3) [9], [16], [19]. This similarity of AP incidence confers our results with some extent of generalizability. However, the studies conducted using the UK medical databases in primary care settings reported a relatively lower incidence of AP for both DM and non-DM subjects [17], [18], [20]. The differences in AP incidence rates among the studies may be related to multiple factors, including the differences in source population, sampling method, disease definition, data collection methodology, analytical methods, and medicosocial factors such as available diagnosis and treatment modality, insurance systems, cultural habits, and hereditary predisposition. Our study population consisted exclusively of patients undergoing any kind of treatment at hospitals for secondary medical care. High frequencies of numerous comorbidities might predispose these patients to developing AP (Table S1, S2) and result in the higher incidences in both the DM and non-DM cohorts. Similarly, our estimates of AP incidence rates were higher than the estimates for the general population in the Japanese national survey in 1998 (IR 20.5 per 100,000 person-years for men, 10.6 for women, including recurrence) [21]. Therefore, these differences in the incidence rates of AP could be partly ascribable to the differences in source populations in different medical practices. Another possibility is that an analysis based on claims data may tend to overestimate disease incidence since medical claims for AP have inevitably been issued when performing tests to rule out AP [9]. To diminish this overestimation, we restricted eligible AP cases for analysis to those patients hospitalized for three days or longer.

Effects of age and gender on AP incidence rates were pronounced regardless of DM status. Male patients were more prone to develop AP, consistent with previous findings. In our non-DM subjects, AP incidence increased as age advanced, as reported in the previous studies [9], [18], [19]. The incidence of AP in our DM cohort also increased as age advanced, which is similar to the age-dependent pattern of AP incidence reported in the Japanese national survey [5], but the previous findings that a higher incidence was reported for the younger generation of patients with DM were not replicated [9], [18], [19]. Although the reason for this lower incidence of AP in our younger patients with DM is unknown, it may be attributable to the relatively shorter follow-up obtained from those patients or specific to Japanese DM population. The differential risks by gender and age observed in the cohort substudy justified a separate, matched case-control analysis for the valid estimation of AP risks by DM and other comorbidities.

A moderate increase in AP risk associated with type 2 DM was confirmed before and after controlling for all the risk factor covariates in the case-control substudy. An approximately two- to three-fold greater risk for patients with type 2 DM was consistently reported regardless of the source population, study sample, geographic location, and race, despite differences in the AP incidence rates observed among the studies (Table S3). In an observational setting, working hypotheses should be tested repeatedly under different situations with various methodologies to draw a solid conclusion. Therefore, our confirmatory, consistent estimates for AP risks should provide robust evidence that type 2 DM is an important risk factor for AP, which has become noted within recent years [9].

Alcoholism, gallstones, and biliary obstruction were also confirmed as independent, strong risk factors of AP, consistent with previous findings [22]. Alcohol and gallstones were reported to be the two most frequent etiologies accounting for 31.4% and 24.4%, respectively, in the Japanese national survey [5]. Because higher frequencies of comorbid risk factors were identified in our case-control substudy rather than the cohort substudy, better sensitivities in detecting comorbid risk factors and a large sample size in the case-control design may have resulted in the higher estimates of AP risks for comorbidities as compared with the previous studies with a cohort-design approach [16], [19]. Further, our multivariate analysis could clearly differentiate increased risks with these known etiologies from potentially false associations with surgeries for digestive system diseases, observed in the univariate model, as confounded by other comorbidities. Because obesity had to be defined by diagnostic codes for morbid obesity, usually requiring medical intervention, we identified the prevalence of morbid obesity at less than 0.2% in cases and controls in the case-control analysis and therefore had to exclude obesity from the model. Hypertriglyceridemia is one of the known etiologies of AP and its causal association with AP was reported above the serum triglyceride level of 1,000 mg/dL [23]. However, dyslipidemia was consistently and significantly associated with a decreased risk of AP in our study. The definition of dyslipidemia in this study may have included variations of lipid abnormalities that were too wide to effectively define the population at potential risk. Otherwise, concomitant antihyperlipidemic agents such as fibrates and statins used in patients with dyslipidemia and insulins and insulin-release agents used in patients with DM may have controlled causal hypertriglyceridemia to such low levels that they do not trigger pancreatitis [23], [24]. In fact, lower frequency of dyslipidemia was seen in the AP cases with type 2 DM compared with the control peers in the case-control substudy (18.0% vs. 39.9%) (Table 3). The Taiwan study reported no significant increase in patients with DM with comorbid hypertriglyceridemia (adjusted HR 1.45, 95% CI 0.60–3.49), suggesting a possible interaction between the presence of DM and comorbid hypertriglyceridemia for AP risk [19]. Additionally, the increased risk by comorbid hypertriglyceridemia was not observed in the US claims database study [16]. Hyperlipidemia reportedly accounted for only 1.4% of etiologies for AP in the Japanese national survey [5]. However, given the difficulties in controlling the complex influences on lipid metabolism of insulin, insulin-releasing stimulants, and other concomitant multiple medications used for DM patients in clinical practice and in defining appropriate thresholds of duration and level of lipid abnormality for AP risk, we believe that it would be impossible to estimate the AP risk with hypertriglyceridemia alone in our observational study.

Drug-induced AP is a known etiology, but is observed only in rare occasions [25]. The Japanese national survey found that drug-induced AP accounted for only 0.5% of its etiologies [5]. Drug-induced AP has been documented primarily based on a case-based approach, in which rather weak causality can be inferred, but has been poorly documented in analytical epidemiological contexts [25], [26]. Additionally, the involvement of antidiabetic agents in AP risks is a point of controversy and some inconsistent findings have been reported [16], [19], [27]–[33]. The eligible population in this study consisted of patients undergoing any kind of medical intervention. Therefore, the risk estimation of AP for patients with DM in the present study should naturally involve the effects of antidiabetic medications. Given this fact, we believe that the complex, evolving treatment regimens using various agents for DM management in clinical practice and the lack of patients' adherence data would make an accurate estimation of AP risk by specific antidiabetic agent almost impractical in this observational setting. Thus, a robust estimation of AP risks associated with the use of particular drugs would require large-scale, prospectively- planned, placebo-controlled clinical trials.

Several other limitations in our study warrant mentioning. The administrative database does not provide several demographic variables such as weight, status of smoking, and alcohol consumption. Therefore, controlling and estimating for these variables is impossible. The disease definitions primarily relied on the records of diagnostic codes and procedures in the hospital administrative database for billing for provided medical care, not for research purposes. Disease ascertainment would not be free from misclassification; however, we used the same conservative algorithm for identifying patients with type 2 DM as used in the previous report [9]. Further, the present study shares the essential limitations of hospital-based researches. Data capture of relevant medical services for a patient may have been incomplete as the nature of hospital-based researches. Therefore, underreporting of diagnoses and a resulting misclassification of exposure and outcome may have occurred and the presence of recurrent AP may be missed in some patients.

The strengths of our study rely on the study design and setting. As AP is a rare clinical entity to be treated at secondary medical care hospitals with inpatient facilities, where the setting of this study was based, efficient case identification and detailed case examination with expertise are expected, indicating a feasible setting for the capture of AP cases and data related to comorbidities [34]. Because of the hospital-based nature, the observed incidence rates of AP may approximate the incidences perceived by practitioners in clinical setting. Further, observed higher incidence rates of AP in the cohort analysis were likely non-differential across the study cohorts such that elevated risk estimates of AP associated with type 2 DM in our study seem valid. Whilst the cohort analysis aimed at estimating crude AP incidence rates, the separate case-control analysis using the same data source provided a larger number of cases for valid risk estimation for DM and multiple comorbidities with exact matching and adjustment.

In summary, despite their confirmatory nature, our risk estimates compatible with the previous findings seem to provide evidence robust enough to establish the universal finding that patients with type 2 DM are at increased risk of AP regardless of geographic location and population. Although DM-associated AP risks had been unknown until recently, this recognition among practitioners is of importance for appropriate clinical management of DM and AP.

## Supporting Information

Table S1 Distribution of diagnosis in ICD-10 classifications in DM cohort and non-DM cohort in the study population.
(DOC)

Table S2 Distribution of diagnosis in ICD10 classifications in acute pancreatitis cases and controls in case-control substudy.
(DOC)

Table S3 Comparison of risk estimates for acute pancreatitis with diabetes and other comorbidities across studies.
(DOC)
