# Supplementary material for: Increased Risk of Acute Pancreatitis in Patients with Type 2 Diabetes: An Observational Study Using a Japanese Hospital Database
Source: PLoS One. 2012 Dec 27;7(12):e53224. doi: 10.1371/journal.pone.0053224 (PMC3531339; doi:10.1371/journal.pone.0053224)
Supplement: Table S1 — Distribution of diagnosis in ICD-10 classifications in DM cohort and non-DM cohort in the study population. (DOC) [file pone.0053224.s001.doc]

Table S1. Distribution of diagnosis in ICD-10 classifications in DM cohort and non-DM cohort in the study population.

| **Blocks** | **Title** | **Type 2 DM cohort (%)** | **Non-DM cohort (%)** |
| --- | --- | --- | --- |
| Total |  | 14,707 (100.0) | 186,032 (100.0) |
| A00-B99 | Certain infectious and parasitic diseases | 5,725 (38.9) | 40,576 (21.8) |
| C00-D48 | Neoplasms | 3,493 (23.8) | 33,646 (18.1) |
| D50-D89 | Diseases of the blood and blood-forming organs and certain disorders involving the immune mechanism | 4,019 (27.3) | 20,033 (10.8) |
| E00-E90 | Endocrine, nutritional and metabolic diseases | 14,707 (100.0) | 38,834 (20.9) |
| F00-F99 | Mental and behavioral disorders | 2,849 (19.4) | 17,156 (9.2) |
| G00-G99 | Diseases of the nervous system | 6,503 (44.2) | 38,591 (20.7) |
| H00-H59 | Diseases of the eye and adnexa | 6,470 (44.0) | 29,936 (16.1) |
| H60-H95 | Diseases of the ear and mastoid process | 1,230 (8.4) | 12,970 (7.0) |
| I00-I99 | Diseases of the circulatory system | 11,558 (78.6) | 54,234 (29.2) |
| J00-J99 | Diseases of the respiratory system | 8,191 (55.7) | 61,846 (33.2) |
| [K00-K93](http://apps.who.int/classifications/apps/icd/icd10online2003/navi.htm#k00) | Diseases of the digestive system | 11,464 (77.9) | 86,510 (46.5) |
| L00-L99 | Diseases of the skin and subcutaneous tissue | 5,736 (39.0) | 41,196 (22.1) |
| M00-M99 | Diseases of the musculoskeletal system and connective tissue | 7,760 (52.8) | 63,994 (34.4) |
| N00-N99 | Diseases of the genitourinary system | 5,882 (40.0) | 48,278 (26.0) |
| O00-O99 | Pregnancy, childbirth and the puerperium | 63 (0.4) | 8,470 (4.6) |
| P00-P96 | Certain conditions originating in the perinatal period | 11 (0.1) | 858 (0.5) |
| Q00-Q99 | Congenital malformations, deformations and chromosomal abnormalities | 485 (3.3) | 2,941 (1.6) |
| R00-R99 | Symptoms, signs, and abnormal clinical and laboratory findings, not elsewhere classified | 8,139 (55.3) | 56,687 (30.5) |
| S00-T98 | Injury, poisoning and certain other consequences of external causes | 5,333 (36.3) | 46,934 (25.2) |
| V01-Y98 | External causes of morbidity and mortality | 1 (0.0) | 10 (0.0) |
