# Supplementary material for: Increased Risk of Acute Pancreatitis in Patients with Type 2 Diabetes: An Observational Study Using a Japanese Hospital Database
Source: PLoS One. 2012 Dec 27;7(12):e53224. doi: 10.1371/journal.pone.0053224 (PMC3531339; doi:10.1371/journal.pone.0053224)
Supplement: Table S2 — Distribution of diagnosis in ICD10 classifications in acute pancreatitis cases and controls in case-control substudy. (DOC) [file pone.0053224.s002.doc]

Table S2. Distribution of diagnosis in ICD10 classifications in acute pancreatitis cases and controls in case-control substudy.

|  |  | **Acute pancreatitis cases** | | **Matched controls** | |
| --- | --- | --- | --- | --- | --- |
| **Blocks** | **Title** | **Type 2 DM (%)** | **Non-DM (%)** | **Type 2 DM (%)** | **Non-DM (%)** |
| Total |  | 244 (100.0) | 1,128 (100.0) | 629 (100.0) | 4,840 (100.0) |
| A00-B99 | Certain infectious and parasitic diseases | 141 (57.8) | 454 (40.2) | 306 (48.6) | 1,707 (35.3) |
| C00-D48 | Neoplasms | 113 (46.3) | 334 (29.6) | 209 (33.2) | 1,174 (24.3) |
| D50-D89 | Diseases of the blood and blood-forming organs and certain disorders involving the immune mechanism | 137 (56.1) | 376 (33.3) | 237 (37.7) | 859 (17.7) |
| E00-E90 | Endocrine, nutritional and metabolic diseases | 244 (100.0) | 576 (51.1) | 629 (100.0) | 1,857 (38.4) |
| F00-F99 | Mental and behavioral disorders | 77 (31.6) | 224 (19.9) | 142 (22.6) | 790 (16.3) |
| G00-G99 | Diseases of the nervous system | 128 (52.5) | 423 (37.5) | 322 (51.2) | 1,565 (32.3) |
| H00-H59 | Diseases of the eye and adnexa | 87 (35.7) | 166 (14.7) | 334 (53.1) | 1,370 (28.3) |
| H60-H95 | Diseases of the ear and mastoid process | 15 (6.1) | 59 (5.2) | 71 (11.3) | 587 (12.1) |
| I00-I99 | Diseases of the circulatory system | 189 (77.5) | 555 (49.2) | 545 (86.6) | 2,393 (49.4) |
| J00-J99 | Diseases of the respiratory system | 165 (67.6) | 530 (47.0) | 413 (65.7) | 2,503 (51.7) |
| [K00-K93](http://apps.who.int/classifications/apps/icd/icd10online2003/navi.htm#k00) | Diseases of the digestive system | 244 (100.0) | 1,128 (100.0) | 549 (87.3) | 3,159 (65.3) |
| L00-L99 | Diseases of the skin and subcutaneous tissue | 131 (53.7) | 366 (32.4) | 313 (49.8) | 1,753 (36.2) |
| M00-M99 | Diseases of the musculoskeletal system and connective tissue | 148 (60.7) | 493 (43.7) | 394 (62.6) | 2,428 (50.2) |
| N00-N99 | Diseases of the genitourinary system | 117 (48.0) | 369 (32.7) | 296 (47.1) | 1,624 (33.6) |
| O00-O99 | Pregnancy, childbirth and the puerperium | 0 | 48 (4.3) | 1 (0.2) | 145 (3.0) |
| P00-P96 | Certain conditions originating in the perinatal period | 0 | 7 (0.6) | 0 | 12 (0.2) |
| Q00-Q99 | Congenital malformations, deformations and chromosomal abnormalities | 15 (6.1) | 21 (1.9) | 25 (4.0) | 158 (3.3) |
| R00-R99 | Symptoms, signs and abnormal clinical and laboratory findings, not elsewhere classified | 183 (75.0) | 655 (58.1) | 402 (63.9) | 2,267 (46.8) |
| S00-T98 | Injury, poisoning and certain other consequences of external causes | 102 (41.8) | 346 (30.7) | 250 (39.7) | 1,714 (35.4) |
| V01-Y98 | External causes of morbidity and mortality | 0 | 0 | 0 | 0 |
