# Supplementary material for: Increased Risk of Acute Pancreatitis in Patients with Type 2 Diabetes: An Observational Study Using a Japanese Hospital Database
Source: PLoS One. 2012 Dec 27;7(12):e53224. doi: 10.1371/journal.pone.0053224 (PMC3531339; doi:10.1371/journal.pone.0053224)
Supplement: Table S3 — Comparison of risk estimates for acute pancreatitis with diabetes and other comorbidities across studies. (DOC) [file pone.0053224.s003.doc]

Table S3. Comparison of risk estimates for acute pancreatitis with diabetes and other comorbidities across studies.

| **Study** | **Our study** | **Noel et al** | **Garg et al** | **Lai et al** | **Girman et al** | **Gonzalez-Perez et al** |
| --- | --- | --- | --- | --- | --- | --- |
| Data source | Japanese Hospital Administrative DB | US HMO DB | US Medco National Integrated DB | Taiwan National Claim DB | UK GPRD | UK THIN DB |
| Incidence rate* |  |  |  |  |  |  |
| Type 2 DM | 475 | 422 | 564 | 277 | 65.9 | 54.0 |
| Control | 165 | 149 | 191 | 142 | 22.0 | 30.1 |
| Risk estimates with Type 2 DM**†** |  |  |  |  |  |  |
| Unadjusted | IRR: 2.88 [2.34, 3.55] |  |  | HR: 1.95 [1.70, 2.24] | HR: 2.89 [2.56, 3.27] | IRR: 1.79 [1.48, 2.18] |
| Age, gender-matched | OR: 1.72 [1.46, 2.04] | IRR: 2.83 [2.61, 3.06] | HR: 2.9 [2.5, 3.5] |  | HR: 2.07 [1.82, 2.34] | OR: 1.67 [1.36, 2.05] |
| Adjusted | OR: 1.86 [1.51, 2.29], HR: 2.30 [1.83, 2.89] |  | HR: 2.1 [1.7, 2.5] | HR: 1.89 [1.65, 2.18] | HR: 1.49 [1.31, 1.70] | OR: 1.37 [0.99, 1.89] |
| Risk estimates with comorbidity**†** |  |  |  |  |  |  |
| Dyslipidemia | OR: 0.62 [0.48, 0.79] |  | HR: 1.4 [0.9, 2.0] ^a^ | HR: 2.39 [1.24, 4.62] ^a^ |  |  |
| Alcoholism | OR: 13.40 [4.27, 42.04] |  | HR: 6.2 [4.5, 8.6] ^b^ | HR: 6.05 [3.62, 10.1] | HR: 1.20 [1.10, 1.31] ^c^ | OR: 1.49 [1.02, 2.18] ^d^ |
| Gallstones | OR: 14.29 [11.60, 17.62] |  | HR: 2.6 [1.9, 3.5] ^e^ | HR: 3.78 [2.77, 5.15] | HR: 1.56 [1.26, 1.93] ^f^ |  |
| Biliary obstruction | OR: 19.23 [11.55, 32.04] |  | HR: 1.4 [0.9, 2.3] ^g^ |  |  |  |
| Other pancreas disease excluding pancreatitis | OR: 1.99 [1.13, 3.51] |  | HR: 24.7 [18.4, 33.3] ^h^ |  |  |  |
| Surgery for digestive system | OR: 1.02 [0.81, 1.28] |  |  |  |  |  |

DB, database; HMO, health maintenance organization; GPRD, general practitioner research database; THIN, the health improvement network; DM, diabetes mellitus; IRR, incidence rate ratio; OR, odds ratio; HR, hazard ratio.

*Per 100,000 person-years. †Point estimates with 95% CI. ^a^ for hypertriglyceridemia, ^b^ for alcohol abuse, ^c^ for any alcohol use, ^d^ for frequent alcohol use, ^e^ for biliary stone disease, ^f^ for gallbladder disease, ^g^ for cholestatic liver disease, ^h^ for pancreatic disease

References:

1. Noel RA, Braun DK, Patterson RE, Bloomgren GL (2009) Increased risk of acute pancreatitis and biliary disease observed in patients with type 2 diabetes: a retrospective cohort study. Diabetes Care 32: 834-838.

2. Garg R, Chen W, Pendergrass M (2010) Acute pancreatitis in type 2 diabetes treated with exenatide or sitagliptin: a retrospective observational pharmacy claims analysis. Diabetes Care 33: 2349-2354.

3. Lai SW, Muo CH, Liao KF, Sung FC, Chen PC (2011) Risk of acute pancreatitis in type 2 diabetes and risk reduction on anti-diabetic drugs: a population-based cohort study in Taiwan. Am J Gastroenterol 106: 1697-1704.

4. Girman CJ, Kou TD, Cai B, Alexander CM, O'Neill EA, et al. (2010) Patients with type 2 diabetes mellitus have higher risk for acute pancreatitis compared with those without diabetes. Diabetes Obes Metab 12: 766-771.

5. Gonzalez-Perez A, Schlienger RG, Rodríguez LA (2010) Acute pancreatitis in association with type 2 diabetes and antidiabetic drugs: a population-based cohort study. Diabetes Care 33: 2580-2585.
